# Grain iron and zinc content is independent of anthocyanin accumulation in pigmented rice genotypes of Northeast region of India

**DOI:** 10.1038/s41598-024-53534-x

**Published:** 2024-02-19

**Authors:** Smrita Gogoi, Sanjay Singh, B. P. Mallikarjuna Swamy, Priyanka Das, Debojit Sarma, Ramendra Nath Sarma, Sumita Acharjee, Sharmila Dutta Deka

**Affiliations:** 1 Department of Plant Breeding and Genetics, Assam Agricultural University, Jorhat, 785013 India; 2 Department of Agricultural Biotechnology, Assam Agricultural University, Jorhat, 785013 India; 3 Plant Breeding Division, International Rice Research Institute (IRRI), Metro Manila, Philippines; 4 Department of Biochemistry and Agricultural Chemistry, Assam Agricultural University, Jorhat, 785013 India

**Keywords:** Biotechnology, Plant biotechnology, Agricultural genetics

## Abstract

The traditional rice genotypes of Assam are considered to have biological value due to the presence of several bioactive compounds like flavonoids, polyphenols, and anthocyanins, which have antioxidant, anti-cancer, anti-diabetic, and anti-aging properties. The pigmented genotypes are considered to have high iron (Fe) content. However, the effect of Fe and Zinc (Zn) accumulation on anthocyanin content is yet to be studied in pigmented rice of Assam. We studied the Fe, Zn, and anthocyanin content in grains of 204 traditional rice of Assam, which are traditionally preferred for their nutraceutical properties. We performed phenotypic and biochemical compositional analyses of 204 genotypes to identify those having high Fe, Zn, and anthocyanin. We also carried out the differential expression of a few selected Fe and Zn transporter genes along with the expression of anthocyanin biosynthesis genes. Interestingly, all pigmented rice genotypes contained a higher amount of phenolic compound than the non-pigmented form of rice. We found the highest (32.73 g) seed yield per plant for genotype Jengoni followed by Kajoli chokuwa and Khau Pakhi 1. We also listed 30 genotypes having high levels of Fe and Zn content. The genotype Jengoni accumulated the highest (186.9 μg g^−1^) Fe, while the highest Zn (119.9 μg g^−1^) content was measured in genotype Bora (Nagaon), The levels of *Ferritin 2* gene expression were found to be significantly higher in Bora (Nagaon) (> 2-fold). For Zn accumulation, the genotype DRR Dhan-45, which was released as a high Zn content variety, showed significant up-regulation of the *ZIP4* gene at booting (> 7-fold), post-anthesis (7.8-fold) and grain filling (> 5-fold) stages followed by Bora (Nagaon) (> 3-fold) at post-anthesis. Anthocyanidin synthase gene, Flavanone 3-dioxygenase 1-like *(FDO1*), and Chalcone-flavanone isomerase-like genes were up-regulated in highly pigmented genotype Bora (Nagaon) followed by Jengoni. Based on our data there was no significant correlation between iron and zinc content on the accumulation of anthocyanin. This challenges the present perception of the higher nutritive value in terms of the micronutrient content of the colored rice of Assam. This is the first report on the detailed characterization of traditional rice genotypes inclusive of phenotypic, biochemical, nutritional, and molecular attributes, which would be useful for designing the breeding program to improve Fe, Zn, or anthocyanin content in rice.

## Introduction

Traditional purple rice genotypes of the Northeastern region of India are referred to as black rice due to the dark pigmentation of the rice grain. The varying pigments are the result of deposits of several compounds in the pericarp layer or aleurone layer. Colored rice varieties or genotypes have long been used in traditional foods of Assam and are considered to have medicinal properties by many cultures in Assam^1^ and other Asian countries^2^. They are also preferred as a natural source of zinc (Zn) and anthocyanin^3–5^. A wide range of variations in the mineral composition and other many bio-active compounds like flavonoids, polyphenols, and anthocyanins was reported in the colored rice of Assam^1^. These bioactive compounds have antioxidant, anticancer, anti-inflammatory, antidiabetic, and anti-aging properties in traditional colored rice^6^. Pigmented rice varieties are also rich in nutrients such as vitamin D, calcium, thiamine, riboflavin, and glutamic acids and are high in fiber, minerals, and protein^7,8^. Anthocyanin pigments help reduce cholesterol levels in humans and are beneficial for diabetic prevention due to the aldose reductase inhibitory activities^1,9^. The pigmented rice varieties are considered to have high biological values and are grouped under functional food ingredients^10^.

Antioxidants in food help to protect living cells and tissues from oxidative damage^11^. Antioxidants, including phenolic compounds, have been especially rich in pigmented rice (black or red pericarp)^11^. Anthocyanin has been identified as the primary phenolic component with anti-oxidative properties in pigmented rice^12^. Dietary increased oxidative stress may be a small but substantial component of the disease reported in dietary Zn deficiency, as evidenced by increased susceptibility to oxidative damage in membrane fractions from specific tissues.

The accumulation of anthocyanin in the rice grains of rice germplasm from different countries has a wide range of genotypic variations^13–15^. The purple rice of Southern Thailand has 9.8 to 245.4 mg 100 g^−1^ of anthocyanin^16^ with high levels of phenol, flavonoids, and antioxidant properties in the pericarp^17^. Moreover, several nutrients such as K, Fe, Zn, Cu, Mg, and Mn are present in the purple rice genotypes^18^.

Genotypic variation has been reported among the local purple rice varieties from northern Thailand grown by the local farmers and is generally consumed as unpolished rice with the pericarp intact without a polishing process^19^. Recently, it has been identified that the local pigmented rice varieties contain high nutritional qualities that are valuable for farmers and consumers such as Fe, Zn, anthocyanin, and antioxidant capacity^20–22^. These are the resources of potential value-adding traits for rice breeders and market benefits as special-quality rice. Therefore, these varieties need to be preserved and promoted through commercialization and public awareness for their therapeutic and nutritional benefits.

Purple rice with pigmented grains is referred to as black rice in the area where very dark pigment is common and the color is formed by deposits of several compounds located in the pericarp layer, seed coat, or aleurone layer. Purple rice has long been a unique and traditional food and medicinal plant in many cultures^2^, as a natural source of Zn and anthocyanin^3–5^. Purple rice contains secondary compounds such as anthocyanins, flavones, tannins, phenolic acids, sterols, tocols, γ-oryzanols, amino acids, and essential oils; among which anthocyanins account for more than 95%^10,23^. China is recognized as the richest in purple rice resources (62%), followed by Sri Lanka (8.6%), Indonesia (7.2%), India (5.1%), Philippines (4.3%), Bangladesh (4.1%), and lesser numbers in Malaysia, Thailand and Myanmar^24^. Currently, the standard classification of purple rice in global markets is similar to that for non-colored rice, mostly involving grain appearance as the main criterion, e.g., size and uniformity of color^5^. Thus, the functional properties will be applied to distinguish the quality of rice grains, including grain nutrients (e.g., Zn and Fe) and anthocyanins that will determine the market value. Earlier it was reported that anthocyanin content and genes involved in anthocyanin production have been linked to Fe concentration^25^.

There is no report on Fe, Zn, and anthocyanin profiling of various colored rice germplasm of the NE region of India. Therefore, we performed phytochemical profiling of existing germplasm. We characterized colored rice landraces originating from the NE region of India for Fe, Zn, and anthocyanin content. We aim to determine the pattern of anthocyanin biosynthesis to iron and zinc content in colored rice germplasms of the NE region of India.

## Materials and methods

### Materials

A group of 204 rice germplasms was collected from the Regional Agricultural Research Station (Titabar), Regional Agricultural Research Station (Lakhimpur), Indian Institute of Rice Research (IIRR) Hyderabad, and Department of Plant Breeding and Genetics of Assam Agricultural University, Jorhat. The seeds were maintained during this research work following the relevant institutional and national guidelines and legislation. Collected genotypes were grown for the multiplication of grains at the experimental area of the Instructional-cum-Research (ICR) Farm, Assam Agricultural University, Jorhat during *Kharif* (July to October)*,* 2019–2020. The harvested grains were dehusked and unpolished rice grains were subjected to grain element compositional analysis for Fe and Zn by using Atomic Absorption Spectrophotometry (AAS). Brown or unpolished rice grain samples were digested by the nitric acid digestion method^26^. 30 genotypes were selected based on their high Fe and Zn content for further phenotypic characterization and phytochemical analysis during *Sali* or winter (June-December) 2020–2021. Also, 5 genotypes were selected based on having high Fe and Zn content along with checks to study the anthocyanin biosynthesis genes along with Fe and Zn transport-related genes. For sample collection, plants were grown in pots during *Sali* (June–December) 2020–2021.

### Growth condition

For phenotypic characterization 30 genotypes were grown in Instructional-cum Research (ICR) Farm, Assam Agricultural University, Jorhat during *Sali* 2020–2021. We selected five (5) genotypes based on their high Fe and Zn content, to study the anthocyanin biosynthesis genes along with Fe and Zn transport-related genes. For sample collection, seeds were germinated in petri dishes and transplanted to pots during June-December (*Sali* or winter season), 2020–2021. A total of 3 replications per genotype were raised by following the best practices [Package and practices for (*Kharif*) crop of Assam, 2015]. Pots were maintained at the net-house of the Department of Plant Breeding & Genetics, AAU. Leaf samples were collected at two different stages: (a) first at the booting stage, (b) second at the post-anthesis stage i.e. after 10 days of flowering. Also, immature seeds were collected 7 days after post-anthesis.

To ascertain the fertility status of the soil used in the experimental pot, before the application of manures and fertilizers, a representative sample was collected before filling out the pot. Then the soil was dried, ground, and allowed to pass through a 2 mm sieve, and composited for analysis of soil fertility status as given in Supplementary Table S1.

### Phenotypic characterization

Phenotypic observations were made from 10 randomly chosen plants in each plot viz*.,* for days to 50% flowering (day), days to maturity (day), plant height (cm), panicle length (cm), spikelet fertility (%), 1000-grain weight (g), grain length (mm), grain breadth (mm), decorticated grain length (mm), decorticated grain breadth (mm), length: breadth ratio, seed yield per plant (g), biological yield per plant (g) and Harvest index (%).

### Phytochemical analysis

Rice grains were de-husked using a hand de-husker and the brown rice grains were ground to flour for further analysis. The moisture content of the grains was within 10–12% at the time of analysis. For total phenol content and antioxidant activity, rice flour (1.5 g) was extracted (1:20 w/v) at room temperature with 85% aqueous methanol. Anthocyanins were extracted using acidified methanol (0.1 M HCl/methanol 85:15, v/v) with a solvent-to-sample (rice flour) ratio of 10:1. The total phenol content was determined using the Folin–Ciocalteu method^27^. The 2, 2-diphenyl-1-picryl hydrazyl (DPPH) radical scavenging activity or antioxidant activity was measured following a previously reported procedure^28^. To determine total anthocyanins, the spectrophotometric method^29^ was employed.

### Gene expression

Gene-specific primers were designed using the Oligo Perfect Designer software program (www.thermofisher.com/oligoperfect.html) having a GC content of 55–60%, a T_m_ > 50 °C, primer length ranging from 18 to 22 nucleotides along with NCBI Locus ID of each gene is presented in Supplementary Table S2.

Total RNA was extracted from leaf and seed samples of all five cultivars subjected to our study, and then cDNA was synthesized. PCR was carried out in the Applied BiosystemsQuantstudio™ 5 Real-Time PCR System (Applied Biosystems, USA). All quantitative real-time PCR experiments were performed twice using two biological replicates and each reaction was run in triplicate using the designed gene-specific primers. Primers specific for rice *Ubiquitin* 10 gene (AK101547) was used as reference gene. Real-time PCR was done using gene-specific primers of anthocyanin biosynthesis, Zn, and Fe transporter genes. Expression profiles were calculated by the ΔΔCT method^30^.

## Results

**Figure 1:**
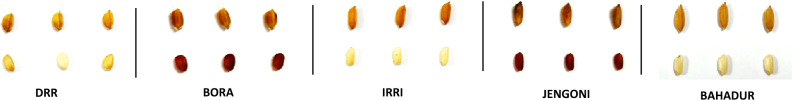
Representative picture of grain samples of the genotypes used in phytochemical analysis and expression analysis.

We categorized all the 204 rice genotypes as low, moderate, and high based on the iron content presented in Supplementary Table S3. Iron content was found highest (186.9 μg g^−1^) in Jengoni followed by IR 95040:12-B-3-10-2-GBS (175.7 μg g^−1^), Herapoa (173.8 μg g^−1^), Doriya (149.7 μg g^−1^), Nagina 22 (149.2 μg g^−1^), Dimrou (145.7 μg g^−1^), Solpuna (140.9 μg g^−1^), Banglami (140.9 μg g^−1^), Krishna (135.8 μg g^−1^), Kmj13A-6-1-2 (129.3 μg g^−1^), Tulashi bora (117.7 μg g^−1^), Katuktara (112.8 μg g^−1^), Kola ahu (103.8 μg g^−1^) and Kajoli chokuwa (102.6 μg g^−1^). However, the highest (119.9 μg g^−1^) Zn content was recorded in Bora (Nagaon), which is one of the glutinous, red-colored genotypes of Assam. We also found high levels of Zn content in Lal Aus (110.2 μg g^−1^), Dikhow (110.1 μg g^−1^), Jyotiprasad (103.1 μg g^−1^), Kmj 13A-6-1-2 (88.2 μg g^−1^), Joha Bora (82.3 μg g^−1^), Solpuna (72.5 μg g^−1^), IR95048:1-B-11-20-10-GBS (72.1 μg g^−1^), Aus Joria (67.4 μg g^−1^) and Nagina 22 (61.7 μg g^−1^). Based on Fe and Zn content; 30 genotypes were selected for phenotypic characterization and phytochemical analysis displayed in Table 1 and Fig. 1a. Out of which 5 genotypes DRR Dhan 45, Bora (Nagaon), Jengoni, IRRI-IR 95040:12-B-3-10-2-GBS, and Bahadur were selected for expression analysis presented in Table 2 and Fig. 1b.

**Table 1:** List of genotypes selected for phenotypic characterization and phytochemical analysis with varied aleurone colour, Fe content* and Zn content*

Sl. no.	Variety name	Aleurone colour	Fe (μg g^−1^)	Zn (μg g^−1^)
1	Bahadur	Light Brown	27.6	22.6
2	Ranjit	Light Brown	56.1	31.2
3	Chittimutylu	Light Brown	61.8	28.4
4	DRR Dhan 45	Light Brown	55.8	32.4
5	Bora (Nagaon)	Red	41.2	119.9
6	Dimrou	Red	145.7	45.5
7	Banglami	Red	140.9	13.6
8	Aus joria	Red	76	67.4
9	Kajoli chokuwa	Red	102.6	30.2
10	Khau pakhi 1	Red	145	20.6
11	Lal aus	Red	28.3	110.2
12	Jengoni	Red	186.9	28.6
13	Tulashi bora	Purple	117.7	46.9
14	Katuktara	Red	112.8	42.9
15	Ikhojoy	Red	74.8	56.6
16	Kola ahu	Red	103.8	40.1
17	Joha bora	White	36.9	82.3
18	Nagina 22	Light Brown	149.2	61.7
19	Krishna	White	135.8	26.3
20	Jyotiprasad	Light Brown	25.2	103.1
21	IR10M210	Light Brown	42.2	57.6
22	Kmj13A-6-1-2	Light Brown	129.3	88.2
23	Solpuna	Light Brown	140.9	72.5
24	Herapoa	Light Brown	173.8	22.8
25	Doriya	Light Brown	149.7	27.1
26	Kobra badam	Light Brown	46	81
27	IR95048:1-B-11-20-10-GBS	Light Brown	46.3	72.1
28	IR 95,040:12-B-3-10-2-GBS	Light Brown	175.7	28.3
29	Dhirendra	Light Brown	69.6	52.9
30	Dikhow	Light Brown	25.2	110.1

*Grains were analyzed at 12% moisture content.

**Table 2:** Genotypes used in expression analysis.

Sl. no.	Genotype	Biochemical composition	Aleurone colour	Fe content (μg g^−1^)	Zn content (μg g^−1^)
1	Jengoni	High iron content	Red	186.9	28.6
2	IR 95,040:12-B-3-10-2-GBS	High iron content	Light brown	175.7	28.3
3	DRR dhan 45	High zinc recommended variety	Light brown	55.8	32.4
4	Bora (Nagaon)	High zinc content	Red	41.2	119.9
5	Bahadur	Control	Light brown	27.6	22.6

### Yield potentials of traditional colored rice genotypes

The presence of variability is a prerequisite for any breeding program. We found significant variation among the colored rice genotypes for yield and component traits presented in Table 3. Dimrou showed early flowering (90 days) and early maturing (106.5 days) genotypes displayed in mean Table 4. However, the highest panicle length (32.38 cm) and plant height (137.97 cm) were observed in Khau Pakhi 1, which is a local traditional germplasm of Arunachal Pradesh. The highest spikelet fertility was observed in the genotype Krishna (89.48%); in which a high Zn breeding line, IR95048:1-B-11-20-10-GBS recorded the highest 1000-grain weight (30.22 g). The highest (32.73 g) seed yield per plant was recorded for Jengoni, which was more than the check variety Bahadur (28.67 g). Other high-yielding genotypes recorded were Kajoli chokuwa (28.25 g), Khau Pakhi 1(26.03 g), Joha Bora (23.38 g), and Herapoa (23.09 g). The highest (55.75%) harvest index was observed for the Kajoli chokuwa genotype.

**Table 3:** Analysis of variance (mean square) for yield related and nutritional traits in rice.

Sources of variation	Degree of freedom	DF	DM	PH	PL	SF	TSW	GL	GW	DGL	DGW	LBR	SYP	BYP	HI	AC
Replications	3	7.63	8.26	5.53	0.72	6.83	3.08	0.09	0.025	0.002	0.004	0.002	13.20	19.83	41.96	2.11
Genotypes	30	182.80	664.62	787.07	17.96	104.09	31.87	1.35	0.147	1.276	0.152	0.545	127.68	380.85	129.97	97.64
Error	90	4.01	3.03	1.60	0.48	1.18	0.48	0.01	0.002	0.008	0.002	0.001	2.84	7.60	12.73	0.32
CV %		1.78	1.19	1.13	2.78	1.31	3.02	1.06	1.55	1.40	1.77	1.28	9.67	6.86	8.36	3.10

*Significant at *P* = 0.05.

**Significant at *P* = 0.01.

*DF* days to 50% flowering, *DM* days to maturity, *PH* plant height, *PL* panicle length, *SF* spikelet fertility, TSW = 1000-grain weight, *GL* grain length, *GW* grain width, *DGL* decorticated grain length, *DGW* decorticated grain width, *LBR* length breadth ratio, *SYP* seed yield per plant, *BYP* biological yield per plant, *HI* harvest index, *AC* amylose content.

**Table 4:** Mean performance of different genotypes for yield related and nutritional traits in rice.

Genotypes	DF	DM	PH	PL	SF	TSW	GL	GB	DGL	DGB	LBR	SYP	BYP	HI	AC
Bahadur	125.00	157.67	100.60	25.13	74.88	18.61	8.51	2.72	6.40	2.34	2.75	28.67	53.19	53.92	22.57
Ranjit	123.67	155.00	92.53	25.11	78.70	18.88	8.26	2.40	6.03	2.03	2.97	26.37	48.04	54.95	20.28
Chittimutylu	108.83	145.83	84.72	23.45	79.14	24.81	7.60	2.80	5.53	2.41	2.30	14.91	32.73	45.70	20.24
DRR Dhan 45	111.17	136.67	112.81	25.37	81.01	23.00	8.55	2.33	6.39	1.95	3.28	15.32	37.33	41.05	21.20
Bora (Nogaon)	111.00	165.00	115.33	22.37	85.35	27.38	9.23	2.85	7.06	2.58	2.67	18.44	45.24	40.77	4.38
Dimrou	90.00	106.50	108.47	23.60	61.79	23.42	8.15	2.82	6.17	2.55	2.42	9.18	21.38	42.93	16.71
Banglami	102.33	126.83	121.20	23.33	89.40	21.29	7.51	2.81	5.63	2.48	2.28	15.54	34.62	44.89	13.86
Ausjoria	119.17	136.83	107.08	25.60	72.55	20.37	7.23	2.93	5.18	2.58	1.99	12.03	31.98	37.59	14.71
Kajoli chokuwa	112.17	157.93	131.25	26.55	82.57	21.04	9.03	2.59	6.91	2.21	3.13	28.25	61.69	45.79	12.23
Khau pakhi 1	116.83	164.33	137.97	31.56	84.66	23.48	8.08	2.31	6.07	1.95	3.08	26.03	55.38	47.01	22.70
Lalaus	113.83	134.17	128.73	27.00	80.45	21.00	8.06	2.58	5.93	2.21	2.67	13.80	25.97	53.16	24.70
Jengoni	100.67	142.33	120.70	24.73	86.27	25.66	9.18	2.92	8.90	2.58	3.50	32.73	58.66	55.75	14.77
Tulashi bora	118.17	168.83	130.07	25.60	81.31	23.27	9.00	2.80	6.80	2.48	2.78	18.76	51.08	36.67	4.55
Katuktara	110.67	134.50	129.33	25.50	87.33	25.68	8.06	2.91	6.05	2.52	2.36	15.65	38.82	40.55	20.71
Ikhojoy	107.00	122.33	112.43	24.08	82.38	21.85	7.70	3.14	5.74	2.80	2.04	9.32	30.25	30.78	19.06
Kola ahu	115.50	134.83	93.37	25.23	84.49	22.79	8.78	2.81	6.75	2.47	2.72	9.26	28.35	32.68	19.79
Joha bora	115.17	161.50	130.70	28.27	86.82	25.92	9.66	2.88	7.56	2.52	2.96	23.38	56.15	41.62	5.45
Nagina 22	113.50	133.83	126.00	25.23	85.04	28.43	8.49	2.91	6.41	2.53	2.52	10.47	33.67	31.12	20.43
Krishna	111.33	132.67	87.30	25.60	89.48	22.87	8.28	2.56	6.17	2.21	2.80	13.61	38.06	35.79	21.26
Jyotiprasad	110.67	160.83	88.58	23.28	85.68	26.02	8.64	2.81	6.61	2.42	2.70	17.58	36.25	30.34	14.00
IR10M210	112.83	163.33	119.53	23.61	81.42	15.03	9.34	2.60	7.19	2.23	3.25	18.40	44.51	41.34	22.11
Kmj13A-6-1-2	105.17	140.33	97.15	22.43	82.96	24.58	9.17	2.81	6.98	2.47	2.83	8.55	32.45	26.22	23.40
Solpuna	106.00	157.67	127.87	26.93	88.10	20.77	8.72	2.95	6.59	2.57	2.53	21.70	49.15	44.17	19.65
Herapoa	127.50	153.67	106.56	19.56	89.43	18.65	7.14	2.82	5.08	2.46	2.07	23.09	55.30	41.74	24.48
Doriya	107.00	151.67	132.00	26.74	89.10	25.98	8.13	2.91	6.06	2.52	2.33	16.45	45.82	35.90	23.85
Kobra badam	121.17	163.50	83.47	24.70	78.21	19.57	9.01	2.61	6.77	2.24	3.06	12.52	40.27	31.10	25.01
IR95048:1-B-11-20-10-GBS	120.50	151.00	95.43	22.85	86.64	30.22	8.42	2.43	6.29	2.09	3.07	14.08	34.49	40.77	19.16
IR 95,040:12-B-3-10-2-GBS	120.83	154.33	107.32	30.77	88.23	22.77	9.01	2.22	6.85	1.89	3.69	9.78	26.72	36.74	19.80
Dhirendra	113.00	150.67	119.03	22.40	86.40	20.55	7.45	2.51	5.37	2.18	2.44	17.85	41.29	43.36	20.69
Dikhow	109.83	140.50	117.00	22.93	84.91	22.64	9.20	2.79	7.22	2.42	3.05	21.46	49.34	43.55	14.91
Mean	112.68	146.84	112.15	24.98	83.16	22.88	8.45	2.72	6.42	2.36	2.74	17.44	48.05	40.93	18.22
SE (m)	0.667	0.580	0.421	0.232	0.362	0.230	0.030	0.014	0.026	0.014	0.017	0.562	0.919	1.189	0.188
CD (*P* = 0.05)	3.303	2.874	2.085	1.146	1.791	1.140	0.148	0.070	0.126	0.069	0.086	2.782	4.550	5.888	0.933
CD (*P* = 0.01)	4.419	3.845	2.790	1.533	2.396	1.526	0.198	0.094	0.169	0.093	0.115	3.722	6.088	7.878	1.248

*DF* days to 50% flowering, *DM* days to maturity, *PH* plant height in cm, *PL* panicle length in cm, *SF* spikelet fertility, *TSW* 1000-grain weight in gm, *GL* grain length in mm, *GW* grain width in mm, *DGL* decorticated grain length in mm, *DGW* decorticated grain width in mm, *LBR* length breadth ratio, *SYP* seed yield per plant in gm, *BYP* biological yield per plant in gm, *HI* harvest index in percentage, *AC* amylose content in percentage.

### Additive gene effects in pigmented rice genotypes

**Figure 2:**
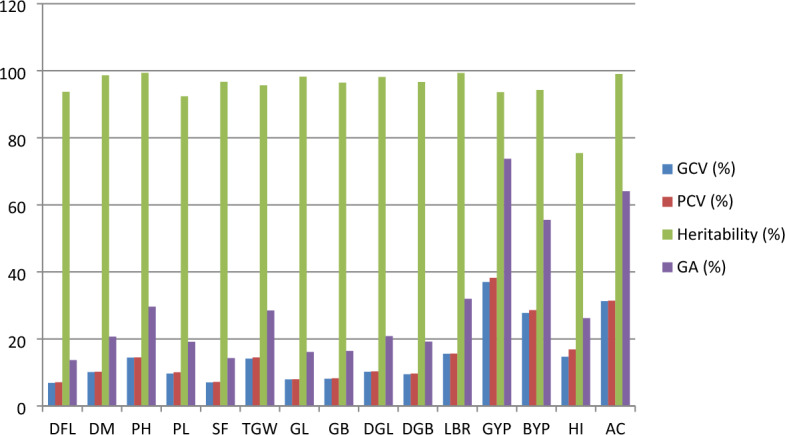
Estimates of genetic parameters for yield and nutritional traits. GCV= Genotypic coefficient of variation, PCV=Phenotypic coefficient of variation, Heritability= Heritability, GA= Genetic advance. Traits are DFL= Days to 50% flowering, DM= Days to maturity, PH= Plant height, PL= Panicle length, SF= Spikelet fertility, TGW= Thousand grain weight, GL= Grain length, GB=Grain breadth, DGL= Decorticated grain length, DGB=Decorticated grain breadth, LBR=Length breadth ratio, GYP= Grain yield per plant, BYP= Biological yield per plant, HI= Harvest index, AC= Amylose content. All the genetic parameters are expressed in percentage (%).

High genotypic and phenotypic coefficients of variation were observed for grain yield per plant, yield per plant, and harvest index. High broad-sense heritability was observed for all the characters presented in Table 5 and Fig. 2. The genetic advance was high for plant height, days to maturity, 1000-grain weight, decorticated grain length, length breadth ratio, seed yield per plant, biological yield per plant and harvest index. Heritability estimates in broad-sense, GCVs, and PCVs were high for grain yield per plant, biological yield per plant, and harvest index. The plant height, days to maturity, 1000-grain weight, decorticated grain length, length breadth ratio, seed yield per plant, biological yield per plant, and harvest index were recorded for high heritability in concurrence with high genetic advance, indicating additive gene effects. Thus, additive gene effects were predominant for these characters in colored rice of the NE region of India.

**Table 5:** Estimates of genetic parameters for various characters for yield related and nutritional traits in rice.

Characters	Range	GCV (%)	PCV (%)	Heritability (%)	GA (%)
Days to 50% flowering (d)	90.00–127.50	6.85	7.08	93.70	13.66
Days to maturity (d)	106.50–168.83	10.11	10.18	98.64	20.69
Plant height (cm)	83.47–137.97	14.43	14.47	99.39	29.63
Panicle length (cm)	19.56–31.56	9.66	10.05	92.35	19.12
Spikelet fertility (%)	61.79–89.48	7.04	7.16	96.68	14.27
Thousand seed weight (g)	15.03–30.22	14.14	14.46	95.63	28.48
Grain length (mm)	7.14–9.66	7.90	7.97	98.22	16.13
Grain breadth (mm)	2.22–3.14	8.11	8.26	96.42	16.41
Decorticated Grain length (mm)	5.08–7.56	10.20	10.30	98.15	20.82
Decorticated Grain breadth (mm)	1.89–2.80	9.48	9.65	96.62	19.20
Length : Breadth ratio	1.99–3.69	15.57	15.62	99.32	31.96
Seed yield per plant (g)	8.55–32.73	36.99	38.23	93.61	73.72
Biological yield per plant (g)	21.38–61.69	27.76	28.59	94.24	55.51
Harvest index (%)	30.78–55.75	14.65	16.86	75.43	26.20
Amylose content (%)	4.38–24.70	31.26	31.41	99.02	64.07

*GCV* genotypic coefficient of variation, *PCV* phenotypic coefficient of variation, *GA* genetic advance.

### High phytochemical activity in pigmented rice genotypes

The estimates of mean square due to genotype were highly significant for all the traits such as total phenol content (TPC), total antioxidant activity, and anthocyanin content indicating sufficient variation among the genotypes studied in Table 6. In our study, all the pigmented rice genotypes contained a higher amount of phenolic compound than a non-pigmented form of rice presented in Table 7. The TPC ranged from 197.74 mg in Lal aus to 554.40 mg in Tulashi bora, which were pigmented rice genotypes. The DPPH (2,2-diphenyl-1-picrylhydrazyl) free radical scavenging activity in brown rice samples ranged from 59.40 to 89.40% in pigmented rice and 9.28% to 16.43% in non-pigmented rice genotypes. Jengoni was recorded to contain the highest antioxidant activity followed by Tulashi bora, Kola Ahu, and Ikhojoy. The anthocyanin content recorded in brown rice samples varied from 57.98–152.07 μg in pigmented rice genotypes. It was observed that in some of the non-pigmented rice genotypes, anthocyanin content was not detected. The highest content of anthocyanin was found in pigmented glutinous rice variety of Assam *i.e.* Tulashi bora (152.07 μg); followed by Kajoli chokuwa (139.87 μg) and Jengoni (127.05 μg).

**Table 6:** Analysis of variance for Phytochemical property.

Source of variation	*df*	Mean squares		
		Total phenol content	Antioxidant activity	Anthocyanin content
Treatment	29	23,322.28**	1658.58**	4710.72**
Error	30	0.63	0.73	15.50
CV%		0.41	2.44	10.04

**Significant at 1% level.

**Table 7:** Mean table for total phenol, DPPH free radical scavenging activity and anthocyanin content of different pigmented and non-pigmented rice germplasm of Assam.

Sl. no.	Variety name	Total phenol content (mg catechol equivalent per 100 g)	DPPH free radical scavenging activity (%)	Total anthocyanin content (ug cyanidin chloride equivalent per 100 g)
1	Bahadur	57.58	12.86	12.08
2	Ranjit	122.06	15.48	5.67
3	Chittimutylu	91.27	12.74	12.08
4	DRR Dhan 45	127.01	16.43	0.00
5	Bora (Nogaon)	262.41	69.64	88.17
6	Dimraw	251.12	55.48	71.01
7	Banglami	262.88	55.83	83.42
8	Ausjoria	278.74	58.93	59.22
9	Kajoli chokuwa	252.52	62.26	139.87
10	Khau pakhi 1	266.14	68.45	57.98
11	Lalaus	197.74	65.24	83.42
12	Jengoni	308.60	89.40	127.05
13	Tulashi bora	554.40	89.05	152.07
14	Katuktara	233.11	54.40	90.45
15	Ikhojoy	368.14	74.52	90.65
16	Kola ahu	343.69	78.21	71.01
17	Joha bora	159.85	9.28	0.00
18	Nagina 22	102.37	11.31	3.39
19	Krishna	93.60	13.93	0.91
20	Jyotiprasad	113.29	13.33	5.46
21	IR10M210	117.95	11.66	0.00
22	Kmj13A-6–1-2	82.49	12.29	0.50
23	Solpuna	98.17	12.02	0.00
24	Herapoa	99.57	12.86	0.70
25	Doriya	169.65	13.93	11.66
26	Kobra badam	135.96	11.67	0.00
27	IR95048:1-B-11-20-10-GBS	124.95	12.62	0.00
28	IR 95,040:12-B-3-10-2-GBS	152.67	12.74	0.00
29	Dhirendra	170.21	13.33	0.29
30	Dikhow	183.09	10.95	9.80
CD 5%		1.15	1.23	5.69
CD 1%		1.54	1.66	7.66
SED		0.56	0.60	2.78

*Analysis were done at grain moisture content 12%.

### Fe and Zn accumulation is independent of coloration

Pearson’s correlation coefficient among the phytochemical traits and micronutrient content mainly, Fe and Zn content were presented in Table 8. A highly significant positive (0.882^**^) correlation was observed between the concentration of TPC and the antioxidant activity in colored rice grains. Also, a positive (0.939^**^) correlation was observed between antioxidant activity and anthocyanin content. A weak positive correlation (0.182) was observed between TPC and Fe content but was statistically not significant; however, a negative correlation was recorded between TPC and Zn content. Our results suggest that there is no direct relation between Zn content and phenol content. Also, there were no significant correlation was observed between Fe and Zn content with antioxidant activity. A positive correlation (0.071) was observed between Fe and Zn content when correlation was studied for 204 genotypes (Supplementary Table S4). However, a negative correlation was observed between Fe content and Zn content in 30 selected genotypes because the selection was based on the levels of Fe or Zn in each genotype. We found that the pigmentation in the colored rice aleurone is not linked with levels of Fe and Zn in rice genotypes used for this study.

**Table 8:** Pearson’s correlation coefficient among the phytochemical traits and micronutrient content.

	TPC	AC	TAC	Fe	Zn
TPC	1	0.882**	0.853**	0.182	− 0.065
AC	0.882**	1	0.939**	0.218	− 0.081
TAC	0.853**	0.939**	1	0.205	− 0.104
Fe	0.182	0.218	0.205	1	− 0.565**
Zn	− 0.065	− 0.081	− 0.104	− 0.565**	1

*TPC total phenol content, *AC* antioxidant activity, *TAC* total anthocyanin content, *Fe* iron content, *Zn* zinc content.

### Transcriptional regulation of Fe, Zn, and anthocyanin content

**Figure 3:**
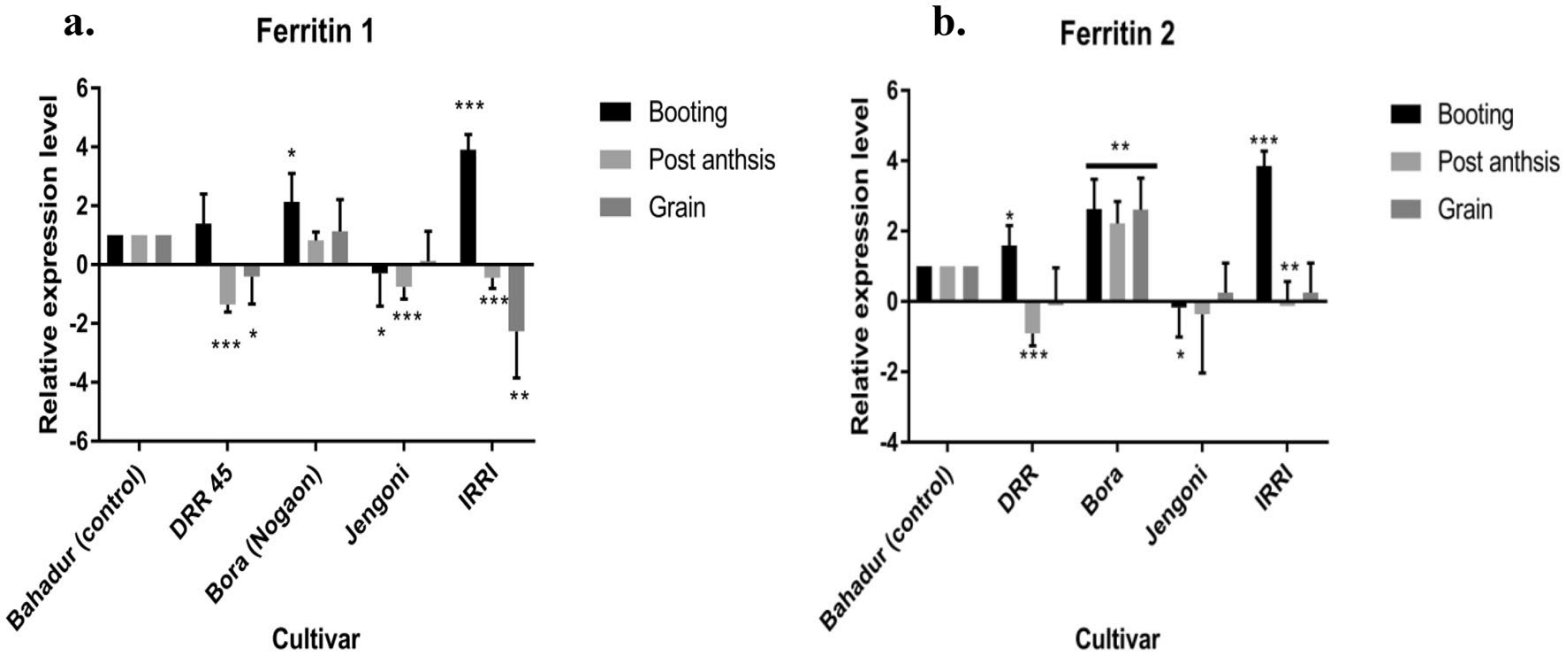
Levels of expression of Fe-regulated genes *Ferritin 1* (**a**) and *Ferritin 2* (**b**)in rice at various stages of growth. an Expression of *Ferritin 1* gene. Expression of *Ferritin 2* gene. Ubiquitin 10 was used as reference gene and Bahadur genotype was used as control. The y-axis indicates the fold change increase/decrease in the expression level of the genes. The x-axis represents the different genotypes used in study. Bars represent Mean ± SEM. * P-value < 0.05, ** P-value < 0.01 and *** P-value < 0.0001. The different color bars indicate the expression of the genes in different genotypes at various rice stages. Black color bars indicate the expression of genes at booting stage using leaf sample, light grey at post anthesis in leaf sample and dark grey at grain filling stage in grain sample.

The *Ferritin 1* (*Fe 1*) gene (AF519570) is responsible for iron storage and helps to protect plants from oxidative stress. Significantly higher (3.904-fold) levels of expression of this gene were observed at the booting stage in the IRRI line IR 95040:12-B-3-10-2-GBS, however, down-regulation of the *Fe 1* gene was observed at the post-anthesis and grain-filling stage (Table 9 and Fig. 3a). Similarly, *Ferritin 2* (AF519571) gene which is related to Fe regulation and accumulation in plants was significantly higher (3.85-fold) in the IRRI line, IR 95040:12-B-3-10-2-GBS at the booting stage and post-anthesis but not during the gran filling stage (Table 9 and Fig. 3b). Interestingly, in Bora (Nagaon) and DRR Dhan 45 genotypes the levels of the *Fe 2* gene were found to be significantly higher at the booting stage [Bora (Nagaon); 2.63-fold and DRR Dhan 1.59-fold]. In the case of DRR Dhan 45, the levels of expression of *Fe 2* were found to be lower compared to the controls at the booting and post-anthesis stages. At the grain-filling stage up-regulation of the *Fe 2* gene was found only in Bora (Nagaon), the remaining genotypes showed down-regulation of the gene.

**Table 9:** Mean and standard deviation for different genes in selected genotypes.

Cultivar	Ferritin 1						Ferritin 2											
	Booting		Post anthesis		Grain		Booting		Post anthesis		Grain							
	Mean	Std. deviation	Mean	Std. deviation	Mean	Std. deviation	Mean	Std. deviation	Mean	Std. deviation	Mean	Std. deviation						
DRR Dhan 45	1.391	1.014	− 1.352	0.252***	− 0.408	0.931*	1.595*	0.568	− 0.908***	0.350	0.045	0.918						
Bora	2.131*	0.970	0.820	0.295	1.139	1.078	2.628**	0.851	2.217**	0.629	2.616**	0.898						
Jengoni	− 0.293*	1.115	− 0.759	0.408***	0.129	1.009	− 0.169*	0.839	− 0.366	1.661	0.129	1.010						
IRRI	3.904***	0.524	− 0.443***	0.359	− 2.262**	1.583	3.854***	0.422	0.017**	0.556	0.254	0.845						

Cultivar	Zinc transporter 1 (*OsZIP1*)						Zinc transporter 4 (*OsZIP4*)											
	Booting		Post anthesis		Grain		Booting		Post anthesis		Grain							
	Mean	Std. deviation	Mean	Std. deviation	Mean	Std. deviation	Mean	Std. deviation	Mean	Std. deviation	Mean	Std. deviation						
DRR Dhan 45	− 4.552***	0.722	− 3.053***	0.964	1.145	0.157	7.382***	2.026	7.897***	1.688	5.028***	0.272						
Bora	− 3.204***	0.611	− 1.738*	1.902	1.827	1.091	− 0.046***	0.381	3.193***	0.470	1.793	0.804						
Jengoni	− 3.168***	0.923	− 1.632***	0.408	0.559**	0.210	5.700***	0.526	6.776***	0.190	3.030***	0.308						
IRRI	2.763**	1.075	− 0.020*	0.789	− 0.582***	0.378	7.010***	1.227	6.121***	0.204	3.338***	0.700						

Cultivar	Anthocyanin synthase						Flavanone 3-dioxygenase 1-like						Chalcone-flavononeisomerase-like					
	Booting		Post anthesis		Grain		Booting		Post anthesis		Grain		Booting		Post anthesis		Grain	
	Mean	Std. deviation	Mean	Std. deviation	Mean	Std. deviation	Mean	Std. deviation	Mean	Std. deviation	Mean	Std. deviation	Mean	Std. deviation	Mean	Std. deviation	Mean	Std. deviation
DRR Dhan 45	− 0.865*	1.464	− 1.533**	1.205	− 1.076***	0.799	3.466***	0.581	3.904***	0.950	2.507**	0.693	0.399***	0.151	0.704	0.370	0.495***	0.073
Bora	1.054	1.711	1.351	0.557	− 1.489	3.355	4.159**	1.829	2.363*	0.898	6.302***	0.229	0.401	1.134	1.921**	0.386	2.987**	0.836
Jengoni	− 1.654**	1.336	− 2.133**	1.430	1.054	0.114	2.809**	0.874	3.850***	0.217	5.827***	0.058	2.460*	1.329	3.478***	0.415	− 12.647***	1.116
IRRI	1.972	1.364	− 0.013*	0.955	0.210*	0.545	3.061***	0.556	2.145**	0.476	− 2.194***	0.397	0.457***	0.209	1.033	0.502	0.047	1.469

*P* value > 0.05 = NS, < 0.05 = *, < 0.01 = **, < 0.001 = ***

**Figure 4:**
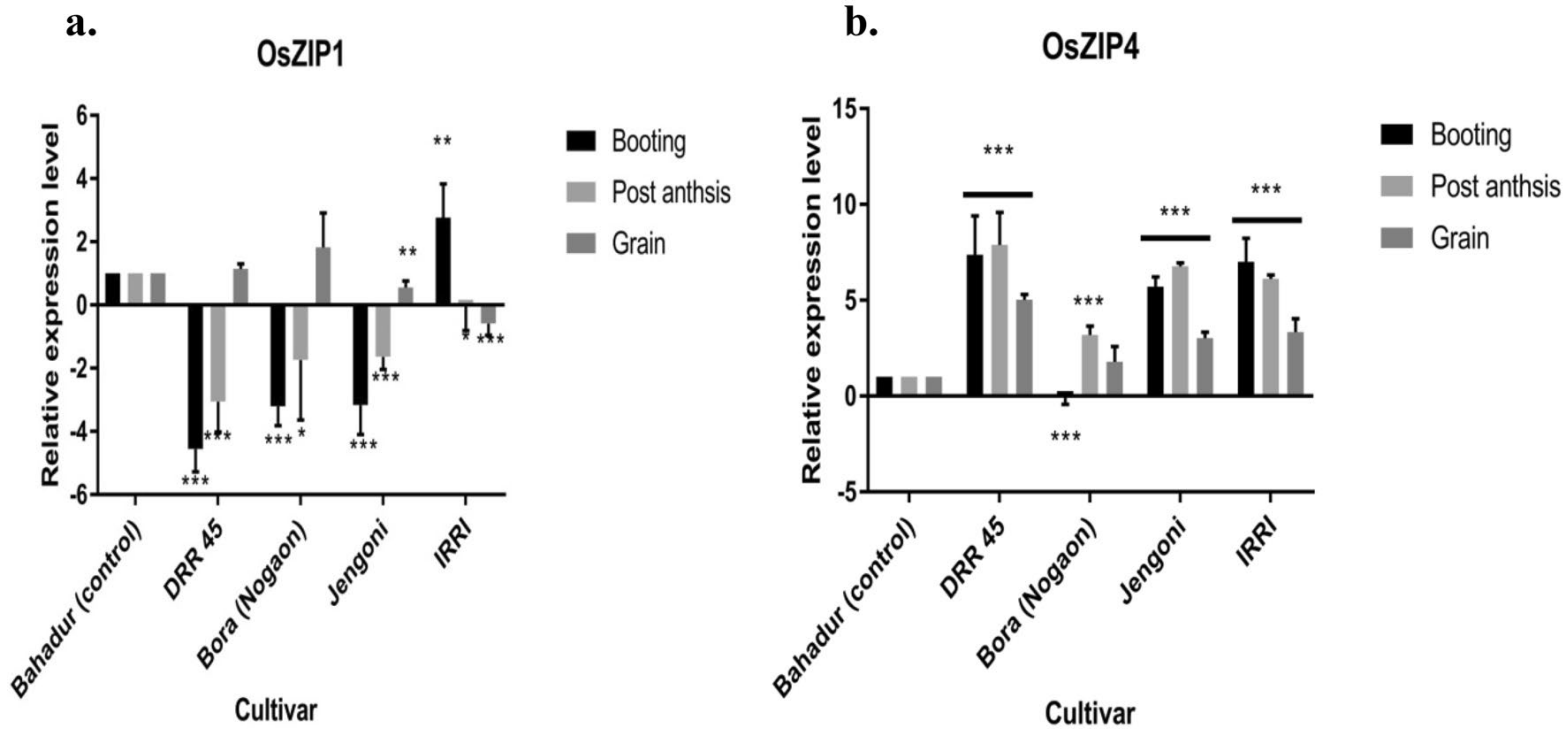
Levels of expression of Zn-regulated genes Zn-regulated transporter-like protein 1(*OsZIP1*; **a**) and Zn- regulated transporter-like protein 4(*OsZIP*; **b**) in rice at various stages of growth. Expression of Zn-regulated transporter-like protein 1(*OsZIP1*) gene. b Expression of Zn-regulated transporter-like protein 4(*OsZIP4*) gene. Ubiquitin 10 was used as reference gene and Bahadur genotype was used as control. The y-axis indicates the fold change increase/decrease in the expression level of the genes. The x-axis represents the different genotypes used in study. Bars represent Mean ± SEM. * P-value < 0.05, ** P-value < 0.01 and *** P-value < 0.0001. The different color bars indicate the expression of the genes in different genotypes at various rice stages. Black color bars indicate the expression of genes at booting stage using leaf sample, light grey at post anthesis in leaf sample and dark grey at grain filling stage in grain sample.

In our study, the expression of Zn transporter-like protein 1(*OsZIP1,* NM_001397949*)* involved in zinc transportation was also studied. The *OsZIP1* (*ZIP1)* gene was found to be downregulated at the booting and post-anthesis stage in both the high zinc-containing variety Bora (Nagaon) and DRR Dhan 45. However, at the grain-filling stage, the levels of expression were found to be up-regulated [Bora (Nagaon); 1.827-fold and DRR Dhan; 1.145-fold] in both the cultivars (Table 9 and Fig. 4a). As expected, the low Zn genotype Jengoni showed down-regulation of *ZIP1* at booting and post-anthesis stages with moderate levels of up-regulation (0.559-fold) at the grain-filling stage. In the case of the IRRI line; IR 95040:12-B-3-10-2-GBS, significant up-regulation was recorded at the booting (2.763-fold) followed by down-regulation at post-anthesis and grain filling stages. Another Zn transporter gene named Zn-regulated transporter-like protein 4(*OsZIP4)* (LOC4344937) showed significant up-regulation in all the varieties at all stages except in Bora (Nagaon) at the booting stage. The variety Bora (Nagaon) showed significant up-regulation of *ZIP4* at the post-anthesis (3.193-fold) and grain-filling stage (1.793-fold). The highest levels of up-regulation of this gene were recorded in DRR Dhan 45 (a high Zn released genotype) with a fold change of 7.382, 7.897, and 5.028-fold at booting, post-anthesis, and grain filling stage, respectively Table 9 and Fig. 4b.

**Figure 5:**
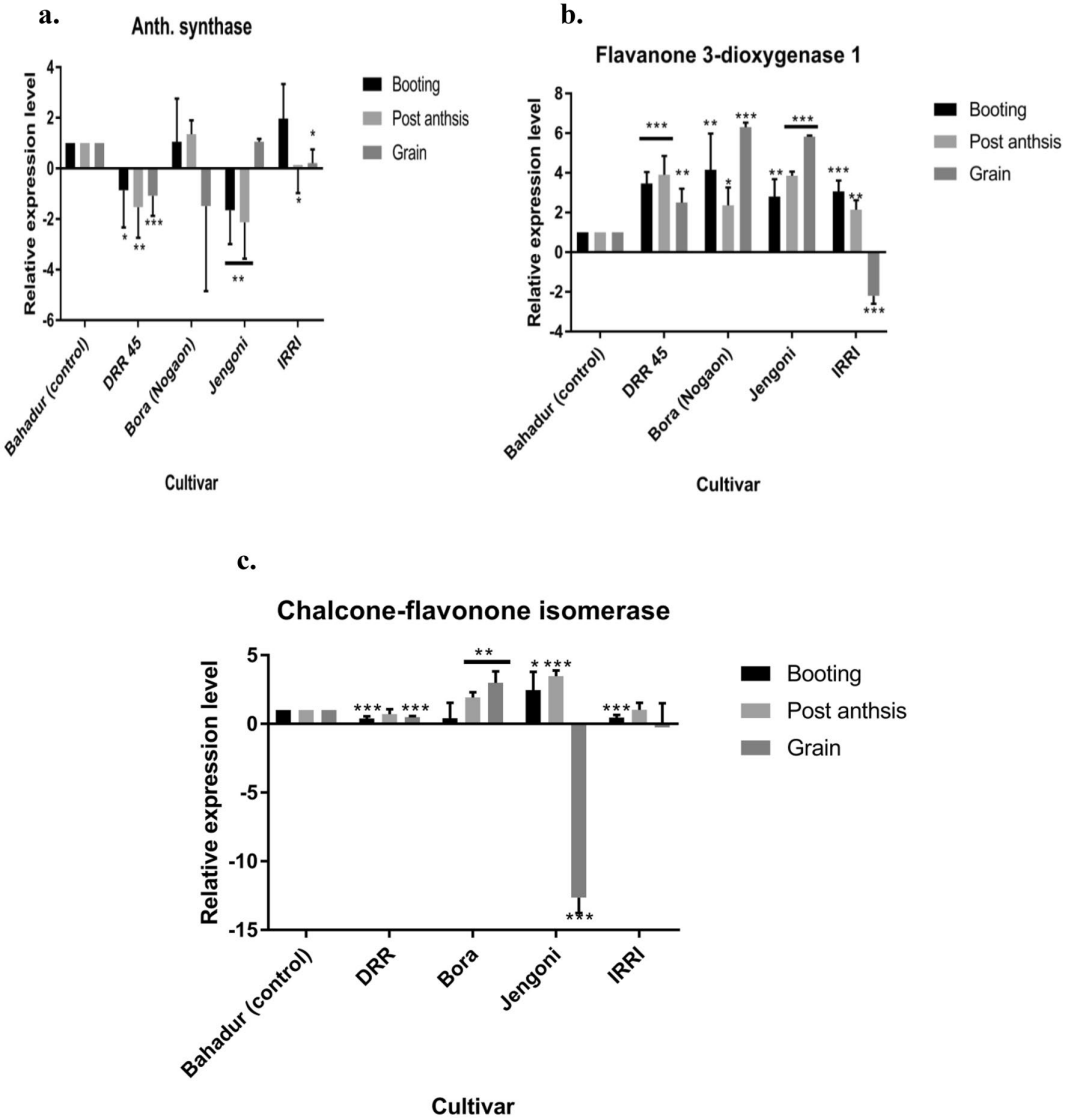
Levels of expression of anthocyanin biosynthesis and accumulated-related genes Anthocyanidin synthase (**a**), Flavanone 3-dioxygenase 1 (**b**) and Chalcone-flavonone isomerase (c) gene in rice at various stages of growth. a Expression of Anthocyanidin synthase gene. b Expression of Flavanone 3-dioxygenase 1 gene and c Expression of Chalcone-flavonone isomerase gene. Ubiquitin 10 was used as reference gene and Bahadur genotype was used as control. The y-axis indicates the fold change increase/decrease in the expression level of the genes. The x-axis represents the different genotypes used in study. Bars represent Mean ± SEM. * P-value < 0.05, ** P-value < 0.01 and *** P-value < 0.0001. The different color bars indicate the expression of the genes in different genotypes at various rice stages. Black color bars indicate the expression of genes at booting stage using leaf sample, light grey at post anthesis in leaf sample and dark grey at grain filling stage in grain sample.

The anthocyanidin synthase gene is responsible for the accumulation of anthocyanin in seeds and other plant parts. *Anthocyanidin synthase* gene (Y07955) was upregulated in highly pigmented varieties, Bora (Nagaon) at the booting and post-anthesis stage, and in the genotype Jengoni, at the grain-filling stage. The biochemical assays also showed high levels of accumulation of anthocyanin in Bora (Nagaon) and Jengoni. In the case of Bora (Nagaon), at the grain-filling stage, this gene was down-regulated although the pericarp showed reddish color deposition Table 9 and Fig. 5a. This implies the development of aleurone color in this genotype may be regulated by other genetic and environmental factors. The flavanone 3-dioxygenase 1-like *(FDO1*) gene (LOC9270463) is reported to be associated with the generation and accumulation of anthocyanins in plants. Significantly higher levels of expression of the *FDO1* gene were recorded in Bora (Nagaon) and Jengoni at different growth stages (Table 9 and Fig. 5b). At the grain-filling stage, Bora (Nagaon) and Jengoni showed about 6.302-fold and 5.827-fold changes in gene expression, respectively. In the Bora (Nagaon) variety, up-regulation (4.159-fold) of the *FDO1* gene was observed at booting followed by post-anthesis (2.363-fold). At the grain-filling stage, a significant (6.302-fold) up-regulation was observed in Bora (Nagaon). Chalcone-flavanone isomerase-like (*CFI)* gene (LOC4334588), an anthocyanin biosynthesis-related gene, was found to be expressed in a highly significant manner in pigmented genotype Bora (Nagaon) at post-anthesis and grain filling stage with a fold change of 1.921 and 2.987, respectively (Table 9 and Fig. 5c). In the case of Jengoni, a higher level of expression of the *CFI* gene is recorded at the booting (2.4-fold) and post-anthesis stage (3.4-fold).

## Discussion

A few accessions of rice maintained at the Directorate of Rice Research (DRR), Hyderabad, India were reported to have the highest concentration of Fe and Zn^31^. In these accessions Fe concentration ranged from 6.2 to 71.6 ppm, while Zn content ranged from 26.2 to 67.3 ppm^31^. Thus, the traditional brown germplasm of Assam might be a good source of breeding material for the biofortification of popular rice cultivars using conventional, acceptable, non-transgenic methods. The nutritive value of rice may vary according to genotypic variation along with several external environmental factors such as soil fertility status, the degree of milling, and the method of preparation before consumption^32^. In our study, Fe content was found highest in the unpolished, brown form of rice seeds of genotype Jengoni, and the highest Zn content was recorded in Bora (Nagaon). Most of the traditional genotypes or landraces are found to have high Fe and Zn content. There is a report on a few indigenous pigmented rice genotypes of Assam containing high Fe and Zn in brown rice form^1^. Other reports are also available on rice genotypes containing high Fe ranging from 6.2 to 186 μg g^−1^ in the grains^31,33–35^. Also, available reports suggested that a few indigenous rice accessions of India have high (> 35 ppm) Zn^31^. Vanlalsanga et al*.*, (2019)^36^ investigated the Zn levels of 65 indigenous rice cultivars from different states in NE India and found that the Badalsali cultivar from Assam had > 75.8 μg g^−1^ Zn, while Manipur's Kapongla accumulated the lowest (17.98 μg g^−1^) Zn in the grains.

There is a common perception among people that pigmented rice genotypes contain higher micronutrients than non-pigmented rice. In our study, we found that Fe and Zn concentrations are not related to the pigmentation, which might be due to the presence of the bran layer in the dehusked grain. Pigmented rice varieties are subject to minimum processing with only pre-cleaning, dehusking, and separation; therefore, bran and germ are left intact^32^. There are various reports on less Zn and Fe content due to prolonged polishing^37,38^. Thus, rice grain Fe content will also vary with the degree of milling/polishing^39,40^. The extent of differential partitioning of Fe and Zn into the panicle and distribution into various grain tissues could be the cause of the variation in the grain Fe and Zn concentrations^37^.

In white rice, nearly 70% of Zn was allocated to the endosperm, compared to only 43% for Fe. Because of the genotypic variation, the degree of milling and partitioning of Fe and Zn into different regions of the grain; milling loss of Fe and Zn might vary. It implied Fe and Zn concentrations in polished rice should not be based entirely on Fe and Zn concentrations in the rice in brown form. Hence, our study implied that with minimum milling during processing and if the aleurone layer is kept intact, it may result in higher nutritional value concerning Fe and Zn irrespective of its aleurone color. Therefore, the common perception of higher micronutrient content in pigmented rice may not always be correct, but it may be more interlinked to the degree of milling. Therefore, the utilization of brown rice in various rice products such as flaked rice popped rice and puffed rice may lead to improvement of Fe and Zn nutrition among the rice-eating population.

We recorded that a few genotypes such as Jengoni, Kajoli chokuwa, Khau Pakhi 1, Joha Bora, and Herapoa had high Fe and Zn content with high phytochemical properties. These genotypes were also found to have high seed yield per plant along with biological yield per plant. Moreover, plant height of genotypes, Kajoli chokuwa, Khau Pakhi 1, and Joha Bora, was also recorded to be taller (131 cm to 137 cm). Therefore, these genotypes can be considered promising genotypes for future breeding programs.

As spikelet fertility is a major determinant of grain yield^41^, there is a significant positive correlation between spikelet fertility with grain yield. We have observed that some traditional *Sali* (winter) genotypes from Assam and *Joha* (aromatic) genotypes have high spikelet fertility. Therefore, the selection of these genotypes with high spikelet fertility could be useful in any breeding program.

The magnitude of phenotypic coefficients of variation was higher than genotypic coefficients of variation for all the traits in our study, indicating that the environment may have an impact on their phenotypic expression^42–45^. However, for all the traits, the difference between GCV and PCV was less, indicating a higher correlation between phenotype and genotype, less environmental effect, and greater importance of genetic factors in quantitative and qualitative trait expression^46^. High estimates of GCV and PCV revealed that all the traits were affected by environmental variation and had a wide range of variability, allowing simple selection for further improvement. High to moderate GCV and PCV indicated the presence of high genetic variability within the genotypes. However, high heritability and genetic advancement would be more useful in selection criteria^47^. Most of the traits were recorded for high heritability in concurrence with high genetic advance, indicating additive gene effects^48^.

Large variability was found for phytochemical content in both pigmented and non-pigmented rice genotypes. We observed similar results as reported in previous studies, which suggest that pigmented rice genotypes contain higher TPC, antioxidant activity (DPPH free radical scavenging activity), and anthocyanin content than non-pigmented ones^17,49–51^. Three times higher total antioxidant activities than the non-pigmented rice were reported by other reports^52,53^. In our study, the genotypes estimated with higher TPC also exhibited high antioxidant activities. Therefore, the study suggested that phenolic compounds including anthocyanins might be primarily associated with the antioxidant activity of rice grains. Also, a highly significant positive correlation was observed in our study between the concentration of total phenolics, antioxidant activity, and anthocyanin in rice grains^1,16,54^. Previous studies reported that the antioxidant capacity of rice increased due to a higher accumulation of anthocyanin^2,55^.

In our investigation, it was recorded that there is no direct significant correlation between the iron and zinc content with other phytochemical content. It implied that phytochemical activities are genotype-specific and the presence of these may be independent of each other. Iron concentration had a considerable negative correlation with rice grain color characteristics and we obtained similar results in our study^16^. Our study also signifies that the nutritive value of pigmented rice genotypes is high relating to higher antioxidant activities but the composition of micronutrients like Zn and Fe may not be sufficiently high. It was reported that the phenolic compounds are mainly associated with the pericarp in rice, however, after milling the concentration of these components in the grain is reduced. Therefore, studying the phenolic and other phytochemical contents in different degrees of polishing of grain and their correlations with different grain layers is crucial. A positive correlation was observed between Fe and Zn content when correlation was studied for 204 genotypes. However, a negative correlation was observed between Fe content and Zn content within 30 genotypes as selection was based on high Fe or high Zn content. Within these 30 genotypes, some genotypes either have high Fe or high Zn, which indicates the presence of component compensation between the two traits^56^. The increase in one trait or component is accompanied by a reduction in other components. Other researchers also observed a positive correlation (0.08) between Fe and Zn for 126 accessions or genotypes^31^.

Plants employ complicated physiological mechanisms at various levels within the rice plant to uptake, mobilize, transport, and load micronutrients into grains. Rice has many genes for micronutrient absorption, translocation, and homeostasis. *Ferritin 1* and *Ferritin 2* are two Fe transporter genes that are active in roots and shoots^57^. Ferritins (particularly *OsFER2*) play a more important role in rice plant growth as part of a defensive mechanism against iron-mediated stress than as an iron-storage protein^58^. As a result, ferritins appear to have no major role as iron-storage proteins in rice grains. In our study, *Ferritin 1* and *Ferritin 2* genes were expressed at high levels in the leaves, mainly at booting stages as expected. It was reported that iron overload resulted in increased mRNA accumulation of both ferritin genes in seedlings and leaves, with *OsFER2* being preferentially elevated^58^. In our study, also the expression of *OsFER2* was higher than *OsFER1* in both the stages of leaves (at booting and post-anthesis). In cultivar Bora (Nagaon), both *Ferritin1* and *Ferritin 2* genes were upregulated during various growth stages. However, biochemical analyses showed a moderate level of Fe in grains. It implied that the genes were expressed but accumulation in the grains was less. The results warrant the involvement of different genes other than the tested ones. However, the presence of gene interaction and change in post-translational modification may be a significant factor in the accumulation of Fe in the matured grain.

Based on the previous reports, it was found that many of the ZIP family genes are triggered when Zn or Fe levels are deficient. *OsZIP1* was discovered to be a rice Zn transporter triggered by Zn deficiency^59–61^. *OsZIP1* is expressed in the vascular bundles of shoots and the vascular bundles and epidermal cells of roots ^59,61^. *OsZIP1* is involved primarily in Zn uptake in roots and Zn homeostasis in shoots^59^. Therefore, down-regulation in high Zn-containing variety at the booting and post-anthesis stage followed a similar trend as reported, previously. However, the present results warrant the inclusion of more pathway genes for Zn accumulation in the rice grain for conclusive remark. Previous researchers have also found that zinc availability, which is controlled by zinc sources, alters the expression patterns of *ZIP1* in rice shoots. Fe sufficiency also lowered *ZIP1* expression in the roots and shoots^59^. However, unlike *ZIP1, ZIP4* expression is not affected by Fe^60,62^. The present study also found that the expression of *ZIP1* was lower than *ZIP4;* which might be due to the Fe sufficiency in the plants.

The development of color in the rice grain is associated with many genetic and environmental factors. Down-regulation of the anthocyanin-related gene in pigmented rice genotypes might be due to their stage-specific expression. It also implies possibilities of association of other genetic and environmental factors in the development of grain color. The role of anthocyanin as an antioxidant and its association with various biotic and abiotic defense mechanisms has been reported in various scientific studies. However, as per our biochemical studies, higher phenol content and antioxidant activities were recorded in the pigmented genotypes Jengoni and Bora (Nagaon). This phytochemical property might lead to the presence of aleurone color in these genotypes. Hence, our study suggested that the color development of rice aleurone is not solely dependent on anthocyanin biosynthesis. There is also no direct association between Fe, Zn content, and aleurone color. They have a different pathway of their own. Reports are also available on the expression pattern of aleurone color which may involve different regulatory genes in the anthocyanin biosynthesis pathway, and it may be genotype-specific^5^. Moreover, tissue-specific expression of the genes with environmental effects may be another cause of the development of color in rice aleurone^5^. It is reported that as the seed matured, the expression of anthocyanin biosynthesis genes increased^63^. There is also a report available that the effect of temperature is one of the causes of the expression of anthocyanin biosynthesis genes^63^. Therefore, it is proposed to evaluate the level of gene expression at different days of interval at the grain filling stages and different temperatures.

### Supplementary Information

Supplementary Figure 1.

Supplementary Tables.

## Data Availability

All data generated or analyzed during this study are included in this published article [and its supplementary information files].
