# Supplementary material for: Grain iron and zinc content is independent of anthocyanin accumulation in pigmented rice genotypes of Northeast region of India
Source: Sci Rep. 2024 Feb 19;14:4128. doi: 10.1038/s41598-024-53534-x (PMC10876706; doi:10.1038/s41598-024-53534-x)
Supplement: Supplementary file 1 — Supplementary Figure 1. [file 41598_2024_53534_MOESM1_ESM.docx]

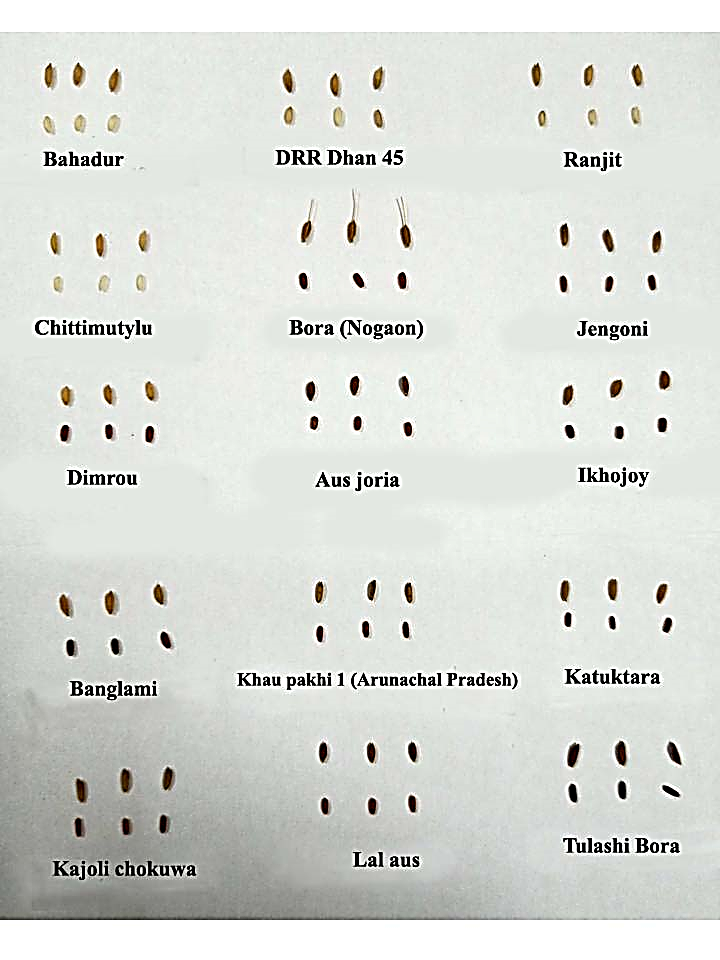


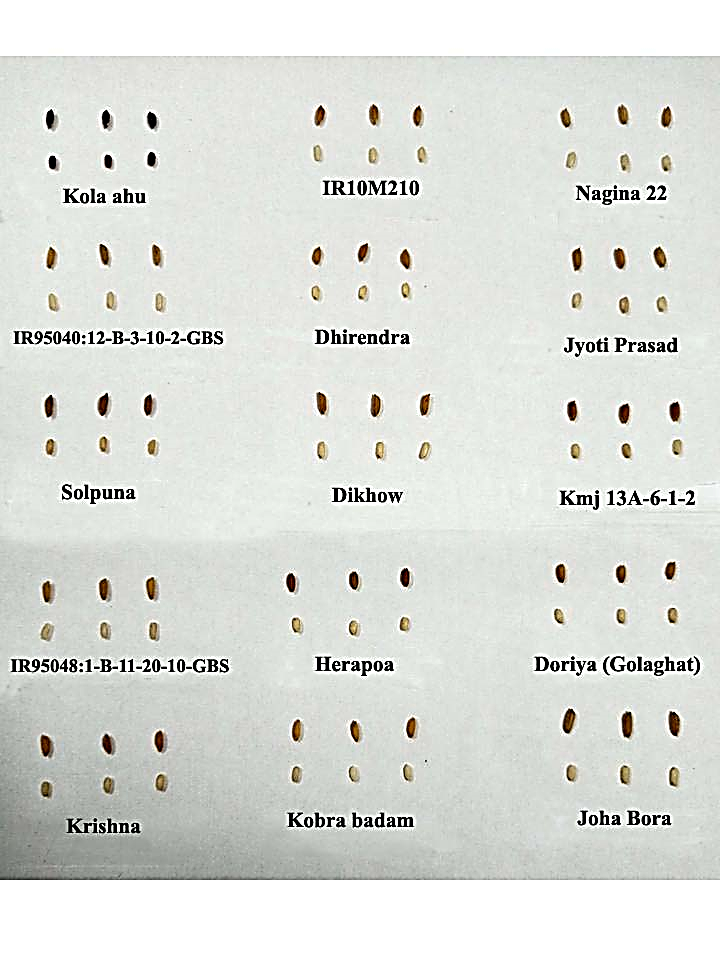


**Supplementary Fig 1:** **Seed phenotype [whole grain (top) and dehusked (bottom)] of 30 genotypes used for phenotypic and biochemical analyses**
