# Supplementary material for: Grain iron and zinc content is independent of anthocyanin accumulation in pigmented rice genotypes of Northeast region of India
Source: Sci Rep. 2024 Feb 19;14:4128. doi: 10.1038/s41598-024-53534-x (PMC10876706; doi:10.1038/s41598-024-53534-x)
Supplement: Supplementary file 2 — Supplementary Tables. [file 41598_2024_53534_MOESM2_ESM.docx]

**SupplementaryTable 1. Soil fertility status of the soil used in pot experiment.**

| **Parameter** | **Value** | **Status** | **Method used** |
| --- | --- | --- | --- |
| pH | 5.23 | Strongly acidic | Glass electrode method |
| Available Nitrogen (kg/ha) | 213.25 | Low | Kjeldahl’s method |
| Available Phosphorus (kg/ha) | 70.55 | High | Bray’s-I method |
| Available Potassium (kg/ha) | 241.92 | Medium | Flame photometric method |
| Available Fe | 21.78 ppm | Medium | Atomic Absorption Spectrophotometer method |
| Available Zn | 0.54 ppm | Low | -do- |

**Supplementary Table 2. List of gene-specific primers used in RT-PCR**

| **Sl. No.** | **Gene name** | **Property** | **Primer sequence** | **Amplicon length (bp)** |
| --- | --- | --- | --- | --- |
| 1 | Ferritin 1  (AF519570) | Leads to iron regulation at the transcriptional level. Also, have roles in iron storage and protection against oxidative stress. | Forward  GGCAGTAGTAGGTTTCGTTTCT | 104 |
|  |  |  | Reverse  CAAATTAAGCACGCAGTAGCAA | 104 |
| 2 | Ferritin 2  (AF519571) | -do- | Forward  CCATCAGCGAGCAGATCAAT | 101 |
|  |  |  | Reverse  TTTGGCGAATCCCTTGAGAG | 101 |
| 3 | Zn-regulated transporter-like protein 1(OsZIP1)  (NM_001397949) | This leads to Zn transportation | Forward  TCGGGCTGCACAAGATATTC | 91 |
|  |  |  | Reverse  TAGACGACGGTCATGAGGAA | 91 |
| 4 | Zn-regulated transporter-like protein 4(OsZIP4)  (LOC4344937) | OsZIP4 is a Zn transporter responsible for Zn translocation to the plant parts that require Zn. | Forward  GCCTGAATACGACCGAAAGT | 90 |
|  |  |  | Reverse  CACACCATTTGCCCAAGAATC | 90 |
| 5 | Flavanone 3-dioxygenase 1  (LOC9270463) | This leads to the generation of anthocyanins and the accumulation of anthocyanins in plants | Forward  CACAGAAGCCCAAGTCTCTC | 82 |
|  |  |  | Reverse  GTCCACAGTACTCCACACATC | 82 |
| **Sl. No.** | **Gene name** | **Property** | **Primer sequence** | **Amplicon length (bp)** |
| 6 | Anthocyanidin synthase  (Y07955) | This leads to the accumulation of anthocyanin in seeds and other plant parts | Forward  CCTCCAGCTCAAGATCAACTAC | 106 |
|  |  |  | Reverse  GTTGTGGAGGATGAAGGAGAG | 106 |
| 7 | Chalcone-flavononeisomerase  (LOC4334588) | Anthocyanin biosynthesis-related genes. Also, OsCHI is a key gene involved in the flavonoid metabolic pathway. | Forward  AAATCGAGCTGCGAATTAACC | 77 |
|  |  |  | Reverse  CAAACACGAGGGCAGTAGAA | 77 |
| 8 | Ubiquitin 10  ([AK101547](https://www.ncbi.nlm.nih.gov/nuccore/AK101547)) | Used as reference gene | Forward  TGGTCAGTAATCAGCCAGTTTGG | 81 |
|  |  |  | Reverse  GCACCACAAATACTTGACGAACAG | 81 |

**Supplementary Table 3. Mean performance of the diverse 204 rice genotypes for Fe and Zn content**

| **Genotypes** | **Fe content (μg g^-1^)** | **Zn content (μg g^-1^)** | **Genotypes** | **Fe content (μg g^-1^)** | **Zn content (μg g^-1^)** | **Genotypes** | **Fe content (μg g^-1^)** | **Zn content (μg g^-1^)** | **Genotypes** | **Fe content (μg g^-1^)** | **Zn content (μg g^-1^)** |
| --- | --- | --- | --- | --- | --- | --- | --- | --- | --- | --- | --- |
| Bor Jahinga | 71.5 | 50.8 | Kurmi Sali | 50.6 | 34.3 | Mala | 64.6 | 44.2 | Gopinath | 97.1 | 37.8 |
| Porabinni | 85.8 | 49.6 | Lalpiki Lahi | 78.8 | 65.4 | Borpeta Local | 30.8 | 15.9 | Meghi | 75.6 | 42 |
| Prafulla | 62.5 | 40.9 | Harkana Sali | 76 | 27.6 | Jengoni | 186.9 | 28.6 | Rangali | 31.7 | 25.9 |
| Idaw (Mizoram) | 77 | 40.4 | Nirmal 1 | 48.1 | 35.7 | Jahinga (Nakachari) | 36.5 | 54.6 | Boka Dhan | 75.3 | 35.9 |
| Mupukliu (Nagaland) | 72.1 | 48.4 | Lahi (Arunachal Pradesh) | 27.1 | 32.3 | Kmj 14S-4-3-4 | 17.9 | 12.1 | Katuktara | 112.8 | 42.9 |
| Kanchatani (Tripura) | 39.2 | 52.1 | Improved Samba Mahsuri | 111.8 | 46.3 | Basmoti Red | 19 | 10.8 | Mitra Sali | 30.9 | 28.3 |
| Kobra Badam | 46 | 81 | Longai | 86.9 | 33.4 | Lewly | 38.6 | 29.2 | Indranarayan | 94.3 | 46.6 |
| Bas | 86.9 | 44.7 | Pankaj | 25.1 | 26.8 | Pyajihari | 107.4 | 47.2 | Joymoti | 40.9 | 21.5 |
| Solpuna | 140.9 | 72.5 | Kmj13A-6-1-2 | 129.3 | 88.2 | Baumurali | 45.3 | 45.1 | Horu Jahinga | 38.7 | 9.5 |
| Betguti 1 | 66 | 43.7 | Sorainokhia | 47.9 | 27.6 | Bormekohi Dhan | 33.2 | 36.9 | Herapoa | 173.8 | 22.8 |
| Suagmoni | 30 | 19.8 | Bora (Sonitpur) | 78.8 | 38.7 | Manuhari (Golaghat) | 90.7 | 23.4 | Bora Dhan | 4.8 | 0 |
| Kolia Krishna | 78 | 26.5 | Basantabahar | 57 | 39.1 | Swarna | 74.3 | 50.8 | Champa | 65.9 | 33.6 |
| Gautam Sali | 26 | 25.1 | Kmj13A-1-3-6 | 128 | 28.5 | Swarna Mahsuri (Dhekiajuli) | 92.4 | 42.6 | Bijoy Mahsuri (Nagaon) | 73.9 | 35.9 |
| Saiamura | 73.7 | 40.2 | Krishna | 135.8 | 26.3 | Dhirendra | 69.6 | 52.9 | Mala Sali (Dhemaji) | 4.7 | 0.5 |
| Bora Chokuwa | 24.9 | 36.5 | Lachit | 55.5 | 50.2 | Paijum (Karbi Angling) | 44.3 | 30.9 | Ronga Sali | 47.8 | 49.9 |
| Dikhow | 25.2 | 110.1 | Muktahar | 43 | 22.3 | Bogi Tora | 103.8 | 20.5 | Sial Sali | 90.1 | 44.1 |
| Chokuwa | 49.9 | 38.2 | Jalashree | 48.4 | 45.2 | Tabaaam (Arunachal Pradesh) | 86.8 | 30.9 | Manohar Sali | 25.5 | 22.8 |
| Red Rice No. 192 | 51.5 | 51.3 | Bahbite (Mizoram) | 92.3 | 45.8 | Sawmliana (Mizoram) | 70.8 | 35.6 | Gemual (Mizoram) | 42.7 | 35.5 |
| Kola Ahu | 103.8 | 40.1 | Kba-le-spah (Meghalaya) | 19.4 | 68.1 | Lahi | 95.3 | 38.9 | Nagina 22 | 149.2 | 61.7 |
| Bora (Jorhat) | 44.8 | 44 | Patal Sali | 19.7 | 25.2 | Dhariya (Chabua) | 23.9 | 17.2 | Bora (Nagaon) | 41.2 | 119.9 |
| Baise | 63.2 | 41.5 | Piolee | 93 | 23.3 | Moniram | 94.6 | 24.1 | Mayamoti | 33 | 16.8 |
| Rajashree | 35.9 | 31.7 | Bordhan-2 | 5 | 13.5 | Betguti 2 | 20.7 | 12.7 | Krishna Ahu | 25.3 | 41.5 |
| Kushal | 78.2 | 39.4 | Kabra Balom | 44.3 | 18.1 | Khaupakhi 1 (Arunachal Pradesh) | 145 | 20.6 | Kolong | 81.5 | 16.5 |
| Doriya (Golaghat) | 149.7 | 27.1 | Khaupakhi (Arunachal Pradesh) | 30.9 | 32.1 | Kopilee | 61 | 47.2 | Horu Begunigootia | 40.5 | 23.6 |
| Lakhimi | 53.2 | 27.6 | Prabhat Sali | 39.9 | 37.2 | Suryamukhi | 85.1 | 52.5 | Koijapuri | 83 | 41.8 |
| Bahadur Check) | 27.6 | 22.6 | Bahadur Check) | 27.6 | 22.6 | Bahadur Check) | 27.6 | 22.6 | Bahadur Check) | 27.6 | 22.6 |
| Ranjit (Check) | 56.1 | 31.2 | Ranjit (Check) | 56.1 | 31.2 | Ranjit (Check) | 56.1 | 31.2 | Ranjit (Check) | 56.1 | 31.2 |
| CH 47 (Check) | 61.8 | 28.4 | CH 47 (Check) | 61.8 | 28.4 | CH 47 (Check) | 61.8 | 28.4 | CH 47 (Check) | 61.8 | 28.4 |
| DRR (Check) | 55.8 | 32.4 | DRR (Check) | 55.8 | 32.4 | DRR (Check) | 55.8 | 32.4 | DRR (Check) | 55.8 | 32.4 |
| **CD (5%)** | **2.11** | **2.23** | **CD (5%)** | **2.11** | **2.23** | **CD (5%)** | **2.11** | **2.23** | **CD (5%)** | **2.11** | **2.23** |

**Supplementary Table 3. contd…**

| **Genotypes** | **Fe content (μg g^-1^)** | **Zn content (μg g^-1^)** | **Genotypes** | **Fe content (μg g^-1^)** | **Zn content (μg g^-1^)** | **Genotypes** | **Fe content (μg g^-1^)** | **Zn content (μg g^-1^)** | **Genotypes** | **Fe content (μg g^-1^)** | **Zn content (μg g^-1^)** |
| --- | --- | --- | --- | --- | --- | --- | --- | --- | --- | --- | --- |
| Banglami | 140.9 | 13.6 | Burali | 16.6 | 30.4 | Black Rice 2 | 76.8 | 36.8 | Bor Malbhug | 59.2 | 36.8 |
| Sadakara | 77 | 27.6 | Haccha | 45.6 | 10.7 | Jul Bao | 25.2 | 31.6 | IR 36 | 57.4 | 47.4 |
| Kokua Bora | 109.8 | 38.1 | Kon Joha 3 | 23.6 | 40.8 | Badal | 32.8 | 22 | IR14M211 | 58.5 | 36.1 |
| IR 64 | 70.7 | 33.5 | Bokul Joha | 76 | 21.7 | Rongi Bao | 74 | 32.2 | Horu Joha | 59.4 | 24.2 |
| Rongdoi | 99.8 | 25.9 | Satyanarayan | 70.2 | 33.8 | Ronga Bao | 38.9 | 19.5 | Badal Sali | 28.8 | 29.9 |
| Kon Joha (Moran) | 26.6 | 26.9 | Basudev | 92.5 | 22.3 | IR10M210 | 42.2 | 57.6 | Ghan Bora | 17.2 | 56.1 |
| Bishnu Prasad | 15.2 | 17 | Negheri Bao | 61.9 | 15.5 | IR15M1298 | 66.5 | 42.8 | Ikhojoy | 74.8 | 56.6 |
| Nirmal 2 | 26.8 | 31.1 | IR15M1341 | 114.3 | 44.8 | IR95048:1-B-11-20-10-GBS | 46.3 | 72.1 | Lal Aus | 28.3 | 110.2 |
| Mulagabhoru | 36.1 | 19.4 | IR14M121 | 15.6 | 43.1 | Maguri | 33.4 | 40.6 | Kajoli Chokuwa | 102.6 | 30.2 |
| Kon Joha 4 | 74.3 | 31.6 | IR 95097:3-B-16-11-4-GBS | 108.3 | 22.8 | Jalkuwari | 48 | 21.9 | Ronga Bordhan | 98.9 | 44.6 |
| IR15M1053 | 29.5 | 33.9 | IR95080:1-B-9-12-17-3 | 42 | 21.5 | Joha Bora | 36.9 | 82.3 | IR 95040:12-B-3-10-2-GBS | 175.7 | 28.3 |
| Mahsuri | 24.2 | 26.1 | IR15M1322 | 46.7 | 17 | Tulashi Bora | 117.7 | 46.9 | Dipholu | 89.9 | 33.4 |
| IR 93354:19-B-12-21-9-1RGA-2RGA-1-B | 29.6 | 21.7 | Indrabhog | 63.3 | 28.3 | Chilarai | 33.7 | 25.5 | Kunkuni Joha | 64.1 | 48.7 |
| IR15M1274 | 37.2 | 30.1 | Kon Joha 5 | 34.5 | 20 | Bali Ghungoor | 48.4 | 38.9 | Tora Sali | 57 | 43.5 |
| IR95052:12-B-3-6-13-B | 39.5 | 47.8 | Keteki Joha | 20.9 | 7.8 | Joha | 63.9 | 35.4 | Baola Bao | 92.3 | 32.2 |
| BPT 5204 | 22.3 | 20.2 | Dimrou | 145.7 | 45.5 | Aus Joria | 76 | 67.4 | Jeera Joha | 41.4 | 39.2 |
| IR 93342:14-B-23-18-5-1RGA-2RGA-1-B | 36.3 | 20.5 | Chokuwa (Pulibor) | 81.9 | 10.8 | Ronga Charmi | 31.8 | 26.2 | Purnendu | 28.8 | 29.9 |
| IR15M1329 | 27.9 | 20.6 | Boka Chokuwa 2 | 92.5 | 31.8 | Joha (Meghalaya) | 74.5 | 52.1 | Kasalath | 74.8 | 39.8 |
| IR 99637-123-1-3-B | 47.6 | 9.7 | Jabbalpur | 38.9 | 23.3 | Black rice 1 | 64.7 | 24.5 | Horu Chokuwa | 20.8 | 28.5 |
| Dehangi | 67.8 | 40.6 | Aghoni Bora | 127.7 | 21.8 | Mou Bora | 102.7 | 29.1 | Dhansiri | 17.5 | 11.2 |
| Kmj 13 AB 1-12-3 | 79.3 | 27.8 | Disang | 22.6 | 28.4 | Buhban | 28.3 | 27.5 | Jaya | 59.5 | 40.6 |
| Mantetoi | 42.4 | 27.3 | Joha (Awn) | 90.5 | 18.6 | Kon Joha | 33.5 | 23.3 | Kanaklata | 50.2 | 47.8 |
| Boka Chokuwa 1 | 84 | 41.4 | Ronga Dhan | 34.8 | 19 | Tandao | 64.4 | 41.9 | Laki | 10.7 | 22.6 |
| Harinarayan | 53.4 | 26.8 | Basundhara | 31.9 | 18.7 | Bezel | 99.1 | 29.7 | Lati Sail | 80.4 | 28.6 |
| Manikimadhuri | 66.6 | 31.6 | Kekua Bao | 98.1 | 31.8 | Jyoti Prasad | 25.2 | 103.1 | Dal Bao | 47.1 | 25.7 |
| Bahadur Check) | 27.6 | 22.6 | Bahadur Check) | 27.6 | 22.6 | Bahadur Check) | 27.6 | 22.6 | Bahadur Check) | 27.6 | 22.6 |
| Ranjit (Check) | 56.1 | 31.2 | Ranjit (Check) | 56.1 | 31.2 | Ranjit (Check) | 56.1 | 31.2 | Ranjit (Check) | 56.1 | 31.2 |
| CH 47 (Check) | 61.8 | 28.4 | CH 47 (Check) | 61.8 | 28.4 | CH 47 (Check) | 61.8 | 28.4 | CH 47 (Check) | 61.8 | 28.4 |
| DRR (Check) | 55.8 | 32.4 | DRR (Check) | 55.8 | 32.4 | DRR (Check) | 55.8 | 32.4 | DRR (Check) | 55.8 | 32.4 |
| **CD (5%)** | **2.11** | **2.23** | **CD (5%)** | **2.11** | **2.23** | **CD (5%)** | **2.11** | **2.23** | **CD (5%)** | **2.11** | **2.23** |

**Supplementary Table 4. Pearson’s correlation between Fe and Zn for 204 genotypes**

| **Character** | **Fe content**  **(μg g^-1^)** | **Zn content**  **(μg g^-1^)** |
| --- | --- | --- |
| Fe | 1 | 0.071 |
| Zn | 0.071 | 1 |
